# Pteridine glycosyltransferase from *Chlorobium tepidum*: crystallization and X-ray analysis

**DOI:** 10.1107/S2053230X17015515

**Published:** 2017-10-30

**Authors:** Asaithambi Killivalavan, Young Shik Park, Kon Ho Lee

**Affiliations:** a Department of Convergence Biomedical Sciences, Graduate School, Gyeongsang National University, Jinju 660-751, Republic of Korea; b Department of Microbiology, School of Medicine, Gyeongsang National University, Jinju 660-751, Republic of Korea; c School of Biological Sciences, Inje University, Kimhae 621-749, Republic of Korea; d Plant Molecular Biology and Biotechnology Research Center (PMBBRC), Gyeongsang National University, Jinju 660-701, Republic of Korea

**Keywords:** pteridine glycosyltransferase, *Chlorobium tepidum*, tetrahydrobiopterin, UDP-*N*-acetylglucosamine, l-*threo*-tetrahydrobiopterin

## Abstract

The pteridine glycosyltransferase (PGT) found in *Chlorobium tepidum* (*Ct*PGT) catalyzes the conversion of l-*threo*-tetrahydrobiopterin to 1-*O*-(l-*threo*-biopterin-2′-yl)-β-*N*-acetylglucosamine using UDP-*N*-acetylglucosamine. The gene for *Ct*PGT was cloned, and selenomethionine-derivatized protein was overexpressed and purified using various chromatographic techniques. The protein was crystallized by the hanging-drop vapour-diffusion method using 0.24 *M* triammonium citrate pH 7.0, 14%(*w*/*v*) PEG 3350 as a reservoir solution. Multiple-wavelength anomalous diffraction data were collected to 2.15 Å resolution from a single *Ct*PGT crystal. The crystal belonged to the monoclinic space group *C*2, with unit-cell parameters *a* = 189.61, *b* = 79.98, *c* = 105.92 Å, β = 120.5°.

## Abstract 2

The cloning, expression, purification, crystallization and preliminary X-ray crystallographic analysis of pteridine glycosyltransferase from *Chlorobium tepidum* are reported.

## 1. Introduction   

Tetrahydrobiopterin (BH_4_) is an essential cofactor for enzymes involved in neurotransmitter biosynthesis, hydroxyl­ation of aromatic amino acids (phenylalanine hydroxylase and tryptophan hydroxylase) and nitric oxide (NO) synthesis (Werner *et al.*, 2011 ▸) in mammals. It also contributes to the proliferation of hematopoietic cells and mammalian cell lines (Thöny *et al.*, 2000 ▸; Nagatsu & Ichinose, 1999 ▸). In addition, BH_4_ may have a role in endothelium-dependent vasodilation in atherosclerosis, diabetes mellitus and vascular dysfunctions of chronic smokers (Hashimoto *et al.*, 2004 ▸). It has been reported that a glycosylated form of BH_4_ is present in some prokaryotes such as *Sulfolobus solfataricus* and *Chlorobium tepidum*, and abundantly in cyanobacteria including *Synechococcus* PCC7942, *Nostoc* sp. and *Synechocystis* sp. (Chung *et al.*, 2000 ▸). In these bacteria, there are a group of enzymes called pteridine glycosyltransferases (PGTs) which catalyse the transfer of sugar moieties from activated donor molecules such as UDP-glucose, UDP-xylose and UDP-galactose to specific pteridine acceptor molecules including BH_4_, biopterin and neopterin to produce various pteridine glycosides (Wachi *et al.*, 1995 ▸).

*Chlorobium tepidum* is a thermophilic, anaerobic phototrophic bacterium. It is one of the primitive model organisms used in the study of photosynthesis. Interestingly, *C. tepidum* possesses a specific BH_4_ stereoisomer, l-*threo*-BH_4_, that differs from the l-*erythro*-BH_4_ (generally known as BH_4_) commonly found in mammals. In addition, a glycosidic l-*threo*-BH_4_, 1-*O*-(l-*threo*-biopterin-2′-yl)-β-*N*-acetyl glucos­amine, exists (Cho *et al.*, 1998 ▸, 1999 ▸). To produce 1-*O*-(l-*threo*-bio­pterin-2′-yl)-β-*N*-acetyl glucosamine, UDP-*N*-acetylglucosamine seems to be used as a donor, providing *N*-acetylglucosamine to the l-*threo*-BH_4_. To date, there is no solid report that explains the catalytic mechanism and formation of the l-*threo*-pteridine compounds with *N*-acetylglucosamine. In *C. tepidum*, there is a gene for pteridine glycosyltransferase (*Ct*PGT; Gene ID 1007245; UniProt ID Q8KE51). In the CAZy database (http://www.cazy.org), the *Ct*PGT protein belongs to the GT-1- and AviGT-4-like protein family.

To understand the structure and mechanism of *Ct*PGT, we isolated the corresponding gene from *C. tepidum* and cloned it for expression in *E. coli*. *Ct*PGT was expressed, purified and crystallized for structural studies. A description of the expression, purification, crystallization and X-ray diffraction analysis of *Ct*PGT is given below.

## 2. Materials and methods   

### 2.1. Macromolecule production   

The gene for pteridine glycosyltransferase from *C. tepidum* (*Ct*PGT) was isolated and amplified by polymerase chain reaction using primers with NcoI and KpnI restriction-enzyme sites (Table 1 ▸; the sites are underlined in the primers). The double-digested PCR product and the pProEX HTa vector (Life Technologies, Carlsbad, California, USA) were mixed in different ratios for ligation at 289 K overnight. The ligated product was then transformed into XL1-Blue cells. Several colonies were selected, and insertion of the DNA fragment was checked by colony PCR and restriction-enzyme digestion with NcoI and KpnI. The insertion of the *Ct*PGT gene into the expression vector was further confirmed by DNA sequencing.

The cloned expression plasmid carrying the *Ct*PGT gene was transformed into *E. coli* strain BL21(DE3) cells for protein expression. An initial culture of 100 ml LB broth (10 g bactotryptone, 5 g yeast extract and 10 g NaCl per litre of solution) with 0.1 mg ml^−1^ ampicillin was seeded with a single colony. This starter culture was incubated at 310 K with vigorous shaking at 180 rev min^−1^ overnight. From the overnight culture, 20 ml of the starter culture was used to inoculate 1000 ml M9 medium (6 g Na_2_HPO_4_, 3 g KH_2_PO_4_, 1 g NH_4_Cl, 0.5 g NaCl, 2 g glucose, 2 m*M* MgSO_4_ and 0.1 m*M* CaCl_2_ per litre of Milli-Q water) in the presence of ampicillin (0.1 mg ml^−1^). All of the essential amino acids including selenomethionine were supplied externally in M9 medium. The cells were then grown at 310 K with shaking at 180 rev min^−1^ until the optical density (OD_600_) reached 0.6, and 0.4 m*M* isopropyl β-d-1-thiogalactopyranoside (IPTG) was subsequently injected to induce protein expression. After adding IPTG, the bacterial culture was shifted to 303 K and grown with shaking at 180 rev min^−1^ overnight. On the morning of the next day, the cells were harvested by centrifugation at 277 K at 6520*g* for 10 min. The pellet was resuspended in 80 ml binding/lysis buffer consisting of 50 m*M* phosphate pH 8.0, 500 m*M* NaCl, 5 m*M* β-mercaptoethanol and disrupted by sonication for 5 min with 3 s pulse and 30% amplitude. It was then centrifuged at 15 930*g* and 277 K for 30 min. The supernatant was collected and filtered using a Whatman No. 1 filter (qualitative filter paper, Advantec, Japan) and applied onto a nickel–agarose (Quiagen, Hilden, Germany) affinity column which had been pre-equilibrated with the binding buffer. The column was then washed with two column volumes of washing buffer which consisted of 50 m*M* phosphate pH 8.0, 500 m*M* NaCl, 5 m*M* β-mercaptoethanol, 30 m*M* imidazole. The bound proteins were eluted with 50 m*M* Tris–HCl pH 8.0, 100 m*M* NaCl, 300 m*M* imidazole, 5 m*M* β-mercaptoethanol.

After elution, fractions containing *Ct*PGT were pooled and the protein solution was exchanged into buffer consisting of 50 m*M* Tris–HCl pH 8.0, 5 m*M* β-mercaptoethanol by ultrafiltration (Centricon YM-30, Millipore Corporation, Bedford, Massachusetts, USA). To cleave the His tag from the protein (the His tag and TEV cleavage site are underlined in Table 1 ▸) the protein was treated with TEV protease (1:20 molar ratio for the protein sample) overnight at 277 K (Table 1 ▸). To separate the cleaved proteins, the reaction mixture was further loaded onto an Ni–NTA column. Flowthrough fractions containing *Ct*PGT were concentrated and injected onto a Mono Q column (GE Healthcare, Piscataway, New Jersey, USA) equilibrated with buffer consisting of 50 m*M* Tris–HCl pH 8.0, 5 m*M* β-mercaptoethanol. The protein was eluted using a salt gradient of 0–0.5 *M* NaCl in the same buffer using an FPLC system (GE Healthcare, Piscataway, New Jersey, USA). The peak fractions were collected and concentrated. Finally, pure *Ct*PGT protein was separated by gel-filtration chromatography using a Superdex 200 column (GE Healthcare, Piscataway, New Jersey, USA) with buffer consisting of 20 m*M* Tris–HCl pH 8.0, 150 m*M* NaCl, 1 m*M* DTT in an FPLC system. All purification steps were performed with ice-cooled buffers at room temperature, which we believe keep the protein stable. The protein purity was checked by SDS–PAGE and native PAGE, and its concentration was determined by the Bradford assay (Zor & Selinger, 1996 ▸; Bradford, 1976 ▸) using bovine serum albumin as a standard.

### 2.2. Crystallization   

Initial crystallization of the selenium-labelled *Ct*PGT was performed with the commercially available screening kits Crystal Screen, Crystal Screen 2 and Index from Hampton Research, California, USA and Wizard Classic 1 and 2 and Wizard Cryo 1 and 2 from Rigaku Reagents, Bainbridge Island, Washington, USA using the microbatch method under Al’s oil in 72-well plates at 291 K. A crystallization drop consisted of 1 µl protein solution (10 mg ml^−1^) and 1 µl screening kit solution. Crystals appeared in 0.2 *M* triammonium citrate pH 7.0, 20%(*w*/*v*) PEG 3350. The hanging-drop vapour-diffusion method was performed to optimize this buffer condition in 24-well cell-culture plates by varying the concentrations of the buffer and precipitant around the condition that produced crystals. Finally, a single crystal that was large enough for diffraction (∼0.4 mm) formed in 4 d using 0.24 *M* triammonium citrate pH 7.0, 14%(*w*/*v*) PEG 3350. The *Ct*PGT crystals were then soaked with 10 m*M* uridine-*N*-acetyl­glucosamine (UDP-NAG) and 5 m*M* dihydrobiopterin (l-*erythro*-BH_2_) for complex preparation. *Ct*PGT was also incubated with 10 m*M* uridine-*N*-acetyl­glucosamine (UDP-NAG) and 5 m*M* dihydrobiopterin (l-*erythro*-BH_2_) in order to grow complex crystals by the co-crystallization method. Crystallization information is summarized in Table 2 ▸.

### 2.3. Data collection and processing   

The crystals were cryoprotected by soaking in solution consisting of 0.24 *M* triammonium citrate pH 7.0, 15%(*w*/*v*) PEG 3350, 25% glycerol. Crystals were scooped out from the cryoprotectant solution using cryoloops and flash-cooled in liquid nitrogen. Multiple-wavelength anomalous diffraction (MAD) data were collected from a single *Ct*PGT crystal on beamline 7A at Pohang Accelerator Laboratory (PAL), Republic of Korea. 360 frames were collected with an oscillation angle of 1° and 1 s exposures with a crystal-to-detector distance of 280 mm. Three data sets were collected at peak (0.979184 Å), edge (0.97934 Å) and remote (0.971549 Å) wavelengths at 100 K. All diffraction images were indexed, integrated and scaled using the *HKL*-2000 suite (Otwinowski & Minor, 1997 ▸). Data-collection details are shown in Table 3 ▸.

## 3. Results and discussion   

**Figure 1:**
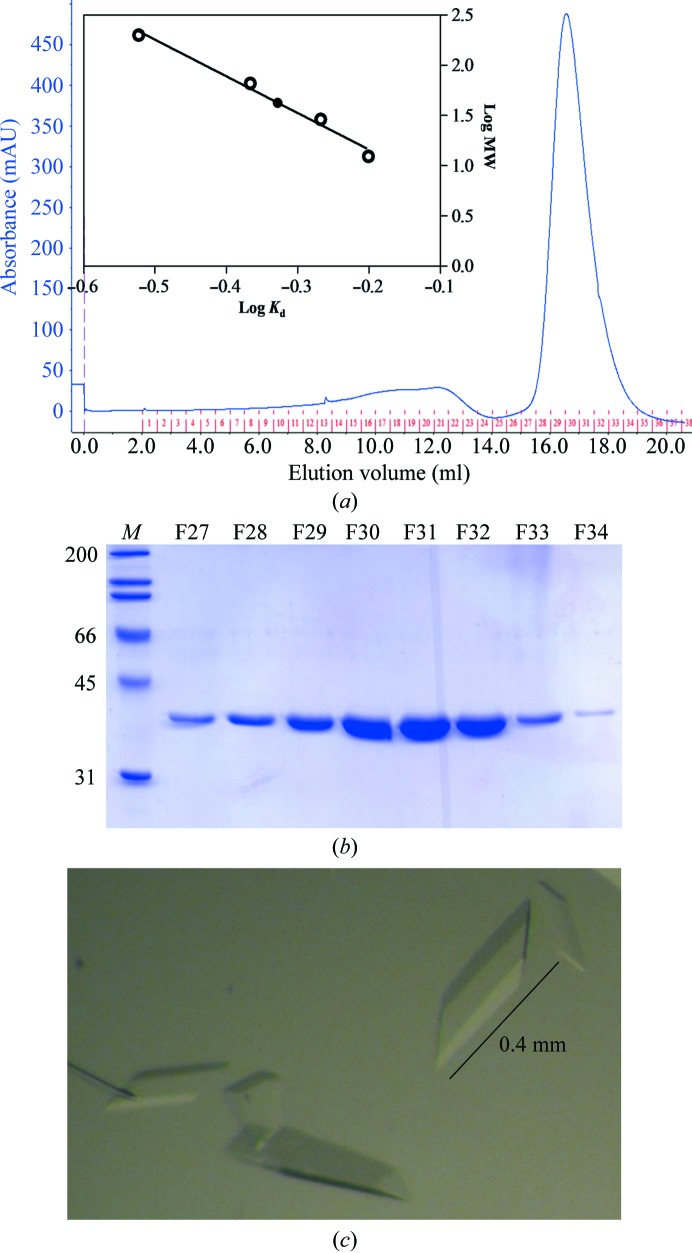
(*a*) Size-exclusion chromatography profile of *Ct*PGT. The molecular size of the *Ct*PGT protein was ∼42 kDa as calculated using protein standards. The graph represents log*K*_d_ plotted against the log of the molecular weights of the protein standards (open circles) and *Ct*PGT (filled circle). *K*_d_ was derived from the equation *K*_d_ = (*V*_e_ − *V*_o_)/(*V*_t_ − *V*_o_), where *V*_e_, *V*_o_ and *V*_t_ represent the elution volume of each standard protein, the void volume and the total volume of the column, respectively. The standard proteins were β-amylase (200 kDa), bovine serum albumin (66 kDa), carbonic anhydrase (29 kDa) and cytochrome *c* (12.4 kDa). (*b*) SDS–PAGE gel of the corresponding peak elution fractions (27–34) shown with markers (labelled in kDa). (*c*) *Ct*PGT protein crystals grown by the hanging-drop vapour-diffusion method. The scale bar represents 0.4 mm.

**Figure 2:**
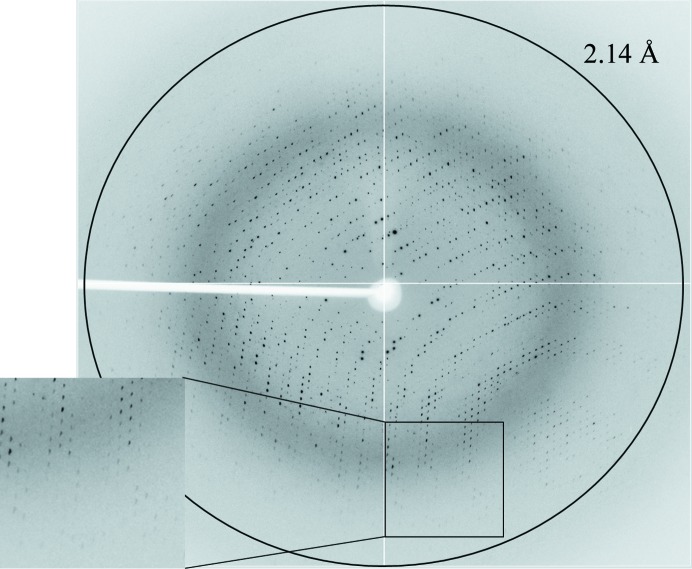
X-ray diffraction image from a *Ct*PGT protein crystal. The black circle represents 2.14 Å resolution.

**Figure 3:**
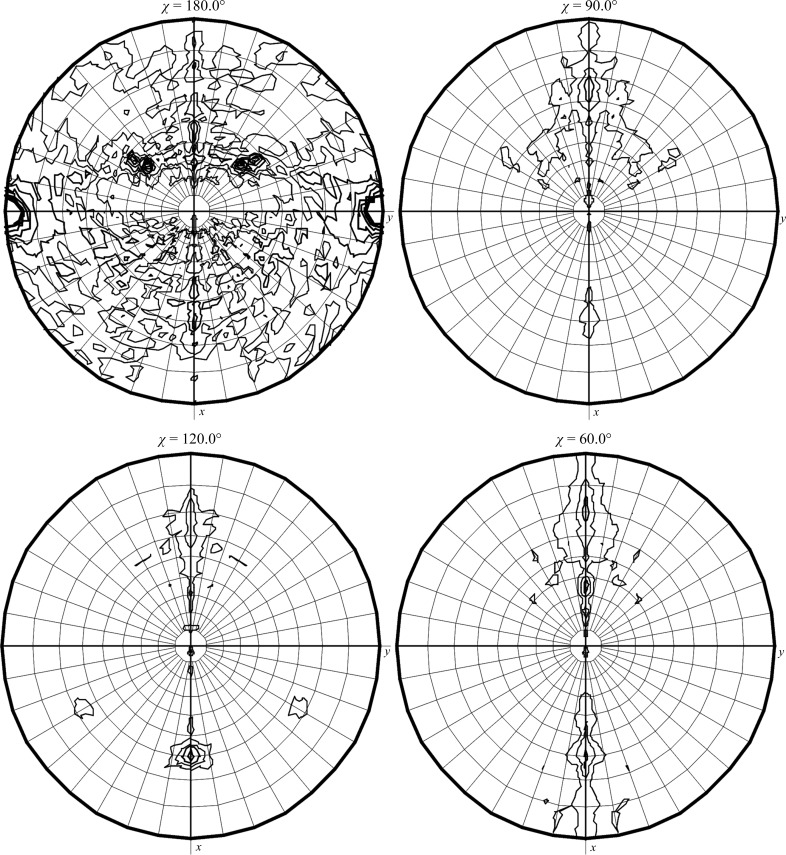
Self-rotation function calculated for *Ct*PGT crystal data, showing the χ = 180° and χ = 120° sections. Peaks are represented as dense contour lines. The calculation was performed using the diffraction data to 3 Å resolution.

**Figure 4:**
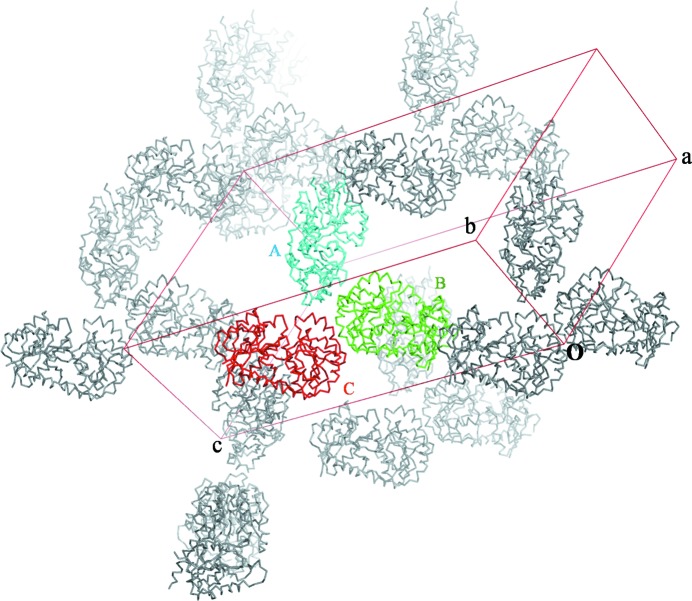
*Ct*PGT crystal packing. The C^α^ chain traces of three molecules are shown in different colours (*A* in blue, *B* in green and *C* in red) in the unit cell (red lines). Other molecules generated by crystal symmetry are displayed in grey.

The gene for *Ct*PGT from *C. tepidum* was successfully cloned in the pProEX HTa expression vector. This plasmid was transformed into *E. coli* BL21(DE3) cells for protein expression. Since *Ct*PGT shares low sequence identity with other glycosyltransferases deposited in the PDB, direct phasing was attempted for structure determination. Therefore, selenomethionine-substituted protein was expressed from *E. coli* BL21(DE3) cells. The expressed protein was soluble and stable. The protein was purified sequentially by nickel-affinity, anion-exchange and gel-filtration chromatography. From the gel-filtration elution profile, the *Ct*PGT protein was found to be a monomer in solution, with an estimated molecular weight of about 42 kDa, which is close to the value calculated from the number of amino acids (356 amino acids; Fig. 1 ▸ *a*). Crystals of *Ct*PGT were obtained in 0.24 *M* triammonium citrate pH 7.0, 14%(*w*/*v*) PEG 3350 (Fig. 1 ▸ *c*). MAD data sets were collected from a *Ct*PGT crystal to 2.14 Å resolution (Fig. 2 ▸) at three wavelengths: peak (0.979184 Å), edge (0.97934 Å) and remote (0.971549 Å). The space group of the crystal was *C*2, with unit-cell parameters *a* = 189.61, *b* = 79.98, *c* = 105.92 Å, β = 120.5°. The Matthews coefficient was 2.92 Å^3^ Da^−1^ (Matthews, 1968 ▸), with a solvent content of 57.89% for three chains, which suggests that there are three molecules in the asymmetric unit. The self-rotation function of the *Ct*PGT crystal data from *MOLREP* (Winn *et al.*, 2011 ▸) clearly showed two prominent peaks at χ = 180°, indicating that there are two noncrystallographic twofold symmetry axes between two molecules among the three molecules in the asymmetric unit. At χ = 120° no peaks were observed for a trimer (Fig. 3 ▸). We determined the *Ct*PGT structure using the peak data by SAD phasing in *PHENIX* (Adams *et al.*, 2010 ▸). MAD was not as successful as SAD. This seems to be because of crystal decay from continuous exposure to radiation, as seen for the data in the remote data set (Table 3 ▸). As a result of *PHENIX* SAD phasing, eight of the nine Se atoms in the molecule were found, which provided a starting electron-density map that was clear enough for model building after density modification. From the C^α^ chain trace, three molecules pack in the asymmetric unit and two pairs among these three molecules are associated by twofold noncrystallographic symmetry, as shown by the self-rotation function (Fig. 4 ▸). The detailed structure will be published soon.

## Figures and Tables

**Table 1:** Macromolecule-production information

Source organism	*C. tepidum*
DNA source	Genomic DNA from *C. tepidum*
Forward primer	5′-TTTCAGGGCGCCATGGATATGAAAAAGCTTAGAATTGCG-3′
Reverse primer	5′-GCCAAGCTTGGTACCCTATGGCAGCGAAGAGAGTGA-3′
Cloning vector	pProEX HTa
Expression vector	pProEX HTa
Expression host	*E. coli* BL21(DE3)
Complete amino-acid sequence of the construct produced	MSYYHHHHHHDYDIPTTENLYFQGAMKKLRIAQVSPLIESVPPKKYGGTERVVYYLTEGLVERGHEVTLFASGDSATSARLIAPVKESLRLGRKIHSTTIMHMLMLSKVYEEMAGEFDIIHSHLEYLTLPYASCSRTPTVLTMHGRLDLPDYADILKRYSSMAWVSISDSQRAPVPDINWVGTIYHGYPENLFEFNPDPEDYFLYLGRFSEEKRPDEAIRLARACKIHLKLAAKIDTADKAYFKAKVEPLLDSPYIEYVGEVGDSRKGELLRNAKALLNTIDWPEPFGLVMIEALACGTPVIVRRCGSSPEVITHGVTGFICDSQLDFIRAIHNIGTISRIACRREFEQRFTLRHMVDNYETLYRKVIAASSATDSLSSLP

**Table 2:** Crystallization

Method	Hanging-drop vapour diffusion
Plate type	24-well plate
Temperature (K)	291
Protein concentration (mg ml^−1^)	10
Buffer composition of protein solution	20 m*M* Tris–HCl pH 8.0, 150 m*M* NaCl
Composition of reservoir solution	0.24 *M* triammonium citrate pH 7.0, 14%(*w*/*v*) PEG 3350
Volume and ratio of drop	1:1
Volume of reservoir (µl)	500

**Table 3:** Data collection and processing Values in parentheses are for the highest resolution shell.

	Peak	Edge	Remote
Diffraction source	Beamline 7A, PAL		
Temperature (K)	100		
Detector	ADSC Q270		
Crystal-to-detector distance (mm)	280		
Rotation range per image (°)	1		
Total rotation range (°)	360		
Exposure time per image (s)	1		
Space group	*C*2		
*a*, *b*, *c* (Å)	189.61, 79.98, 105.92		
α, β, γ (°)	90, 120.5, 90		
Wavelength (Å)	0.979184	0.979340	0.971549
Resolution range (Å)	50–2.15 (2.19–2.15)	50–2.15 (2.19–2.15)	50–2.60 (2.64–2.60)
No. of unique reflections	73216 (3143)	73272 (3391)	42057 (2121)
Completeness (%)	99.2 (86.2)	99.6 (92.6)	100 (100)
Multiplicity	7.3 (5.7)	7.3 (5.9)	7.5 (7.6)
〈*I*/σ(*I*)〉	9.5	9.3	5.6
*R*_r.i.m._ † (%)	12.8 (89.5)	12.27 (92.8)	16.54 (104.8)

† These values of the redundancy-independent merging *R* factor *R*_r.i.m._ are estimated by multiplying the conventional *R*_merge_ value by the factor [*N*/(*N* − 1)]^1/2^, where *N* is the multiplicity.
